# High Satisfaction and Low Distress in Breast Cancer Patients One Year after *BRCA*-Mutation Testing without Prior Face-to-Face Genetic Counseling

**DOI:** 10.1007/s10897-015-9899-4

**Published:** 2015-11-04

**Authors:** Aisha S. Sie, Liesbeth Spruijt, Wendy A. G. van Zelst-Stams, Arjen R. Mensenkamp, Marjolijn J. L. Ligtenberg, Han G. Brunner, Judith B. Prins, Nicoline Hoogerbrugge

**Affiliations:** Department of Human Genetics 836, Radboud University Medical Center, PO Box 9101, 6500 HB Nijmegen, The Netherlands; Department of Pathology, Radboud University Medical Center, Nijmegen, The Netherlands; Department of Medical Psychology, Radboud University Medical Center, Nijmegen, The Netherlands

**Keywords:** BRCA, Breast cancer, Counseling, DNA, Genetic, Hereditary

## Abstract

According to standard practice following referral to clinical genetics, most high risk breast cancer (BC) patients in many countries receive face-to-face genetic counseling prior to *BRCA*-mutation testing (DNA-intake). We evaluated a novel format by prospective study: replacing the intake consultation with telephone, written and digital information sent home. Face-to-face counseling then followed *BRCA*-mutation testing (DNA-direct). One year after *BRCA*-result disclosure, 108 participants returned long-term follow-up questionnaires, of whom 59 (55 %) had previously chosen DNA-direct (intervention) versus DNA-intake (standard practice i.e., control: 45 %). Questionnaires assessed satisfaction and psychological distress. All participants were satisfied and 85 % of DNA-direct participants would choose this procedure again; 10 % would prefer DNA-intake and 5 % were undecided. In repeated measurements ANOVA, general distress (GHQ-12, *p* = 0.01) and BC-specific distress (IES-bc, *p* = 0.03) were lower in DNA-direct than DNA-intake at all time measurements. Heredity-specific distress (IES-her) did not differ significantly between groups. Multivariate regression analyses showed that choice of procedure did not significantly contribute to either general or heredity-specific distress. BC-specific distress (after BC diagnosis) did contribute to both general and heredity-specific distress. This suggests that higher distress scores reflected BC experience, rather than the type of genetic diagnostic procedure. In conclusion, the large majority of BC patients that used DNA-direct reported high satisfaction without increased distress both in the short term, and 1 year after conclusion of genetic testing.

## Introduction

Patients confronted with a diagnosis of breast cancer (BC) desire quick answers about their personal situation in light of their risk of a hereditary predisposition (Salemink et al. 2013). Should a pathogenic *BRCA1/2*-mutation be found, BC patients are at an increased risk of up to 60 % of a second primary BC (Antoniou et al. 2003; Chen and Parmigiani 2007; King et al. 2003) which may influence the choice of BC treatment (Trainer et al. 2010). In addition, these patients are also at a high risk for ovarian cancer (20–60 % for *BRCA1* and 2–20 % for *BRCA2* (Antoniou et al. 2003; Chen and Parmigiani 2007; King et al. 2003)) and prophylactic surgery is recommended (Hermsen et al. 2007). Family cascade screening may identify unaffected *BRCA1/2*-mutation carriers with an increased lifetime risk of 40–80 % for BC (Antoniou et al. 2003; Chen and Parmigiani 2007; King et al. 2003). *BRCA1/2*-mutation carriers are eligible for yearly BC screening or prophylactic surgery from 25 years of age (Kurian et al. 2010). Following referral to clinical genetics of high risk BC patients, current genetic counseling practice in many countries typically involves a face-to-face counseling session with a genetic counselor prior to diagnostic *BRCA*-testing (Balmana et al. 2011; Berliner et al. 2013; Robson et al. 2015; Wham et al. 2010). This may add several weeks to the period of diagnostic uncertainty regarding *BRCA1/2* gene status. We hypothesized that a shorter timeline for providing genetic testing information might be advantageous for BC patients with concerns about their risk of a hereditary predisposition.

To achieve this, we previously evaluated short-term patient experiences with a novel format replacing the initial face-to-face consultation prior to *BRCA*-mutation testing (usual care, DNA-intake procedure) by telephone, written and digital information with a blood drawing kit sent to their home address (DNA-direct procedure) (Sie et al. 2012, 2014a). In both procedures, *BRCA*-results were disclosed in face-to-face consultations by an experienced genetic counselor, including personalized counseling and cancer prevention recommendations for both patients and their families (Sie et al. 2012). Given a free choice between these procedures, 59 % (95 of 161) of eligible BC patients (*p* = 0.03) chose the new format of *BRCA*-mutation testing without prior face-to-face genetic counseling (DNA-direct), indicating an interest in this new procedure. DNA-direct participants were highly satisfied and showed lower psychological distress several weeks (median 5 [2–22]) after *BRCA*-result disclosure than DNA-intake. This suggests that patients with higher distress were more likely to opt for initial face-to-face contact prior to genetic testing and remained more distressed throughout the procedure (Sie et al. 2014a).

While these short-term results were reassuring, literature shows different trajectories of change in psychological adjustment after BC diagnosis: while the majority remains even or stabilizes 1 year post-diagnosis, a small group deteriorates in mental functioning, steadily declining until reaching a plateau at 19 months (Helgeson et al. 2004). This trend was shown in older (>65 years) BC patients, where diminished social support was predictive of deteriorating quality of life (Ganz et al. 2003). BC patients may also be vulnerable due to family cancer history, e.g., deaths of family members diminishing their social support systems. Family history is often the reason for referral to genetic services (Nelson et al. 2014). We therefore sought to determine long-term effects and acceptability of the novel DNA-direct procedure, in order to assess whether distress is triggered at a later time.

This study thus compared long-term experiences of BC patients (satisfaction and psychological distress) between the novel DNA-direct procedure and usual care (DNA-intake), measured 1 year after *BRCA*-result disclosure. We hypothesized that patient satisfaction in both procedures would remain stable over time (as we observed previously shortly after *BRCA*-result disclosure), and that DNA-direct does not induce increased distress in short- or long-term.

## Methods

### Participants

**Fig. 1:**
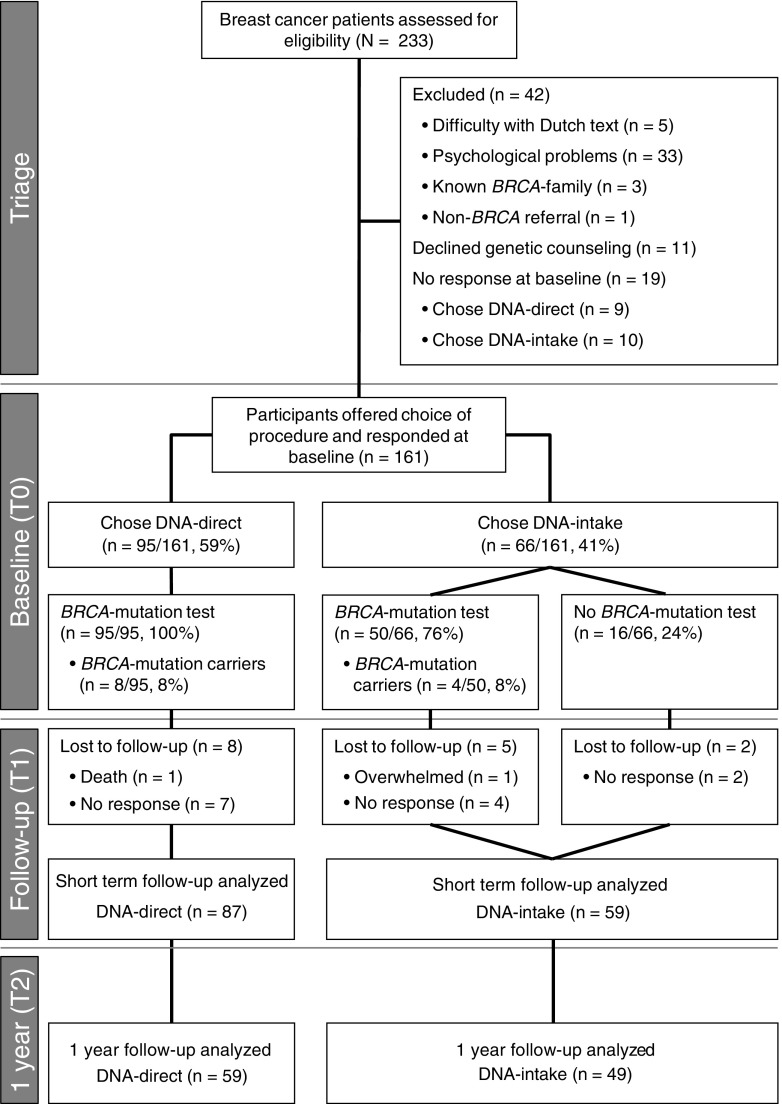
Flowchart of patient inclusion, short term and 1 year follow-up, procedure proportions and *BRCA*-results

The study protocol was previously published (Sie et al. 2012). In short, following approval by the local medical ethics committee, all female patients (previously) diagnosed with BC and referred to the department of Human Genetics at Radboudumc between August 2011 and February 2012 were eligible (Fig. 1). Exclusion criteria were psychological problems requiring treatment, difficulty with Dutch text, or known *BRCA*-families (being associated with different risks of having the known family *BRCA*-mutation, therefore different information to provide and considerations to make). To evaluate whether there was a preference for DNA-direct, BC patients were free to choose between procedures.

### DNA-Direct Procedure

As published previously (Sie et al. 2012, 2014a), in the novel DNA-direct procedure, patients received telephone (triage call by a trained medical doctor), written and digital information (website, educational movie) at home. The triage call (median 9 [5–20] minutes) served to check exclusion criteria primarily meant for pre-test psychosocial assessment of difficulty with Dutch text, psychological problems (i.e., current psychological treatment by counseling and/or medication) or family communication problems (i.e., self-reported need for guidance) (Fig. 1). Non-excluded patients were offered the choice of DNA-direct versus DNA-intake to all participants, without genetic counseling. Patients choosing DNA-direct received an informational letter and website with video covering basic information about BC, heredity and genetic testing, similar to a pre-test consultation. A blood drawing kit was included to start *BRCA*-mutation testing. Written informed consent for DNA-direct and family history forms were required before diagnostic testing was initiated. Telephone or e-mail contact with the physician researcher (AS) was available (used by 14 participants, only regarding logistics). All *BRCA-*results were disclosed in a face-to-face consultation of 45 min (equal to the pre-test DNA-intake consultation) by one of five experienced genetic counselors.

### Study Procedure

Previous results were published (Sie et al. 2014a) based on 161 responses on baseline (T0) questionnaires. Of these, 95 (59 %) chose the DNA-direct procedure over DNA-intake, and 146 (*n* = 87 DNA-direct) returned short-term follow-up (T1) questionnaires sent 2 weeks after *BRCA*-result disclosure. Mutation detection rate was equal at 8 % in both groups; processing time was 1 month shorter in the DNA-direct procedure, hypothesized to be related to the waiting time until the initial appointment for an intake consultation as *BRCA*-mutation testing time did not differ between groups. Additional long-term follow-up (T2) data are presented here, collected from questionnaires sent 1 year after *BRCA*-result disclosure to previous T1 responders; participation was voluntary.

### Instrumentation

The current paper reports new results of 1 year follow-up (T2), in which the primary study outcomes were: satisfaction regarding choice of procedure (T1/T2), general distress (T0/T1/T2: GHQ-12 (Goldberg et al. 1997) scale 0–12, Cronbach’s α in this study = 0.84 at T0 / 0.89 at T1 / 0.88 at T2) and heredity-specific psychological distress (T0/T1/T2: IES-her (Horowitz et al. 1979; van der Ploeg et al. 2004) scale 0–75, α = 0.93 / 0.94 / 0.95).

Secondary psychological measures were BC-specific distress (T0/T2: IES-bc (Horowitz et al. 1979; van der Ploeg et al. 2004) α = 0.93 / 0.92), global quality of life (T0/T1/T2: selected from EORTC-QLQ-Q30 (Bottomley and Aaronson 2007) scale 0–100, α = 0.85 / 0.84 / 0.91), BC worry (T0/T1/T2: CWS (Lerman et al. 1994) scale 8–32, α = 0.84 / 0.85 / 0.82), risk perception of hereditary BC and 2nd BC (T0/T1/T2: visual scales 0–100). Other secondary T2 outcomes were: coping style (shortened TMSI (Ong et al. 1999; van Zuuren et al. 1996)) categorizing responders as more monitoring (subscale α = 0.69) i.e., actively seeking information about medical threats, more blunting (subscale α = 0.69) i.e., seeking distraction, or neutral, as used in a previous study (Sie et al. 2013); and open-ended questions regarding a) perceived causes of their BC and b) most important aspects for other patients to know about genetic testing (>10 % reported).

### Baseline Differences

In previous analyses (Sie et al. 2014a), significant differences in baseline (T0) sociodemographic and BC characteristics were found between DNA-direct and DNA-intake groups (Table 1). Most importantly, DNA-direct participants reported higher website use (*p* = 0.01), more prior information by their referring physician about personal consequences (*p* = 0.004), less prior information by their referring physician about genetics in general (*p* = 0.008) and lower decisional conflict i.e., difficulty making a decision whether to start DNA-testing (*p* = 0.01). Baseline differences were corrected for statistically as described below.

**Table 1:** Relevant baseline differences (*p* < 0.05) in sociodemographic and breast cancer (BC) characteristics for all BC patients choosing DNA-direct (novel format) or DNA-intake (usual care) as evaluated in previous analyses (Sie et al. 2014a)

Characteristic	DNA-direct *n* = 95: N (%) or median [range] or mean ± SD	DNA-intake *n* = 66: N (%) or median [range] or mean ± SD	P
Age at inclusion	49 [23–73]	53 [28–74]	0.10
Age at 1st BC diagnosis	47 [23–71]	49 [28–74]	0.15
Months since last BC	6 [0–247]	6 [0–195]	0.92
*BRCA* referral criteria			
- positive family history	75 (79 %)	53 (80 %)	1.00
- age at BC <40 yrs	29 (31 %)	13 (20 %)	0.15
- ovarian cancer in patient	4 (4 %)	2 (3 %)	1.00
Family characteristics			
- mother with BC	17 (18 %)	13 (20 %)	0.84
- sister with BC	17 (18 %)	15 (23 %)	0.55
- age (yrs) youngest with BC	40 [23–62]	42 [26–64]	0.03 *
- children living at home	55 (58 %)	26 (39 %)	0.03 *
Educational level			
- high	39 (41 %)	13 (20 %)	0.01 *
- medium	27 (28 %)	25 (38 %)	
- low	29 (31 %)	28 (42 %)	
Use of BC websites	50 (53 %)	21 (32 %)	0.01 *
Information provided by referring physician			
- genetics in general	25 (26 %)	31 (47 %)	0.008 *
- personal consequences	39 (41 %)	13 (20 %)	0.006 *
- outcomes of genetic testing	32 (34 %)	12 (18 %)	0.03 *
Decisional conflict (DCS: 0–100)	*n* = 87: 16.2 ± 13.9	*n* = 58: 23.2 ± 10.8	0.001 *

* Statistically significant *p* < 0.05: baseline differences included as covariate in multivariate analyses

### Data Analysis

Data is presented using descriptive statistics. To compare DNA-direct versus DNA-intake for each T2 outcome, the unpaired *t*-test was used for continuous, Mann–Whitney *U* test for ordinal and chi-square/Fisher’s Exact test for nominal/dichotomous variables. To correct for eight baseline differences (Table 1), these were included as covariates in repeated measurements ANOVA used to test for changes over the three time measurements (T0, T1, T2) between DNA-direct versus DNA-intake (group) in psychological outcomes (general distress, heredity-specific distress, BC-specific distress, quality of life, BC worry and risk perception of hereditary BC and 2nd BC). The same correction was performed in multivariate regression analyses of non-psychological outcomes showing univariate differences to determine if such differences persist after correction. Correlations between distress (T2: general, heredity-specific, BC-specific) and choice of procedure (DNA-direct or DNA-intake), sociodemographic characteristics (T0: age at inclusion, educational level), BC characteristics (T0: age at 1st BC diagnosis, months since last BC, *BRCA* referral criteria, family characteristics) and psychological variables (T2: quality of life, coping style, BC worry, risk perception for heredity and for second BC) were assessed using Pearson’s correlation coefficients. Characteristics with significant correlations were used as independent variables in multiple backward linear regression analysis for the determinants of each psychological distress measure. The probability level for statistical significance testing was set at 0.05 (two-tailed). The SPSS 20.0 statistical package was used to analyze the data.

### Genetic Counselors’ Experiences

Previously unreported, the five involved genetic counselors filled in a yes/no checklist after each individual DNA-direct consultation to determine whether they experienced: 1) good rapport with the patient, 2) unexpected patient reactions, 3) need for a follow-up consultation, 4) need for non-standard psychosocial support, 5) the patient having made an informed choice to start *BRCA*-testing, and 6) the retrospective preference for a pre-test intake consultation. They were also asked for their general opinions during a joint DNA-direct counselor meeting.

## Results

A total of 108 BC patients returned 1 year follow-up (T2) surveys of whom 59 had previously chosen DNA-direct (55 %), five of which were identified as *BRCA-*mutation carriers, versus 49 participants who had chosen DNA-intake of which one was a *BRCA*-mutation carrier.

### Satisfaction with Choice of Procedure

All participants in both groups were satisfied with their choice of procedure, 75 % strongly so; none reported regret. Most DNA-direct participants (85 %) would choose this procedure again (one participant emphasized the benefit of taking action from home during BC diagnosis/treatment) whereas 10 % now preferred DNA-intake (one stated it would be more personal to talk to a genetics professional rather than read information) and 5 % did not know (one clarified dependency on their health at that time). In DNA-intake, most (80 %) would choose this procedure again with 10 % emphasizing personal contact, but 16 % now preferred DNA-direct (none clarified) and 4 % did not know (depending on explanation of the procedure).

Two-thirds (63 %) of DNA-direct versus one-third (31 %, *p* = 0.001) of DNA-intake reported that their recommended procedure to another patient would depend on that individual person: their preferences for personal contact, information formats, comfort using digital media, questions and worries, capability of processing information, prior medical knowledge, social support. DNA-direct was specifically recommended by 24 % of DNA-direct and 10 % of DNA-intake participants (one felt the choice could also be made using DNA-direct, one would recommend DNA-intake instead if the person had many worries). DNA-intake was recommended by 9 % of DNA-direct (one mentioned the ability to ask questions) and 57 % of DNA-intake (one emphasized their own preference for face-to-face contact). Five percent of DNA-direct and 2 % of DNA-intake participants were uncertain which procedure to recommend; all stated it was a personal choice.

### Psychological Distress

**Fig. 2:**
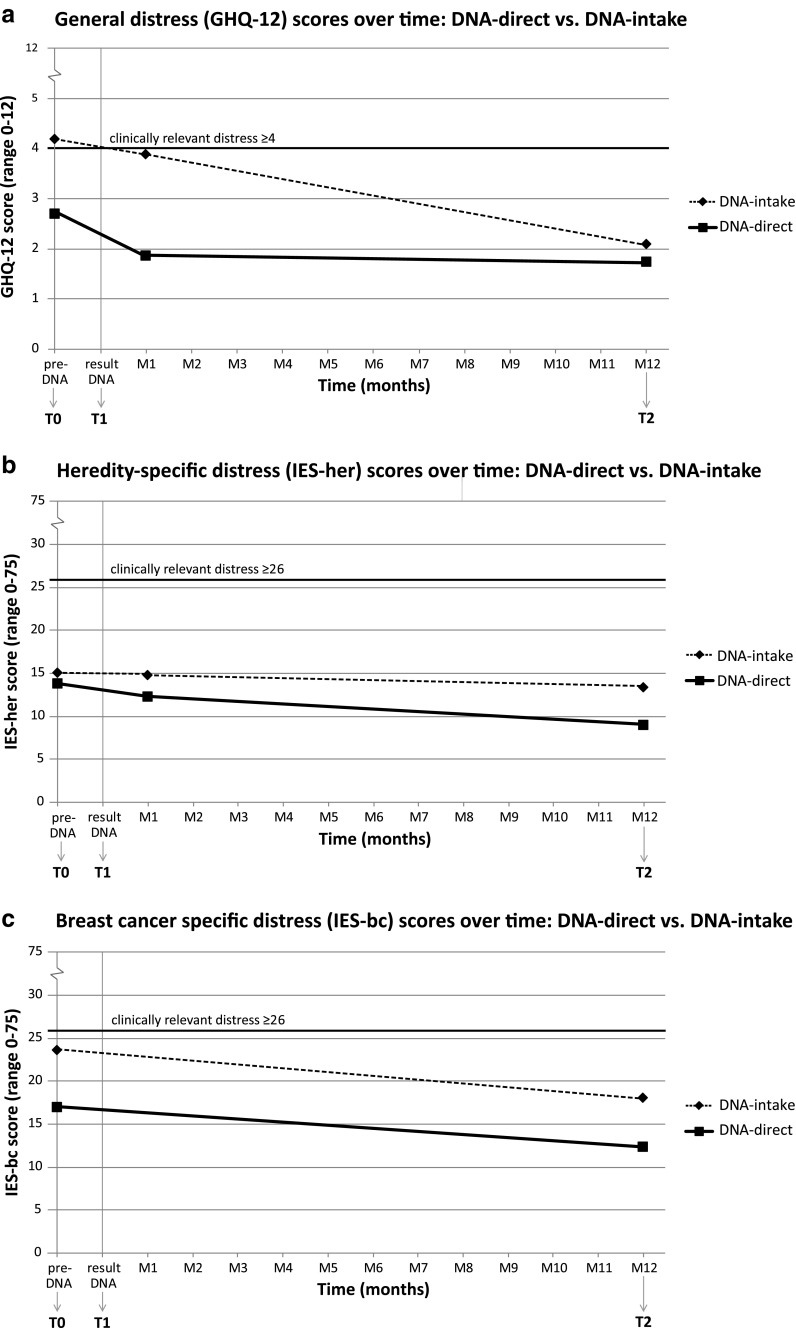
Changes over time in psychological distress measures: **a**) general distress (GHQ-12), **b**) heredity-specific distress (IES-her), and **c**) BC-specific distress (IES-bc). Significant group effects were found only in general distress (**a**) and BC-specific distress (**c**) without significant time effects in any measure

As shown in Table 2, no main effects for time (within subjects) were found for any psychological distress measure. In DNA-direct, lower scores were reported for general distress than DNA-intake (GHQ-12: *p* = 0.01, between subjects). Notably, a near-significant interaction effect between time and choice of procedure was found (*p* = 0.051): as seen in Fig. 2a, the difference in general distress between procedures appears greater at T0 and T1 than at T2. Corrected mean general distress scores for DNA-intake crossed the threshold for clinical relevance of GHQ-12 ≥ 4 at baseline (Fig. 2a) but dropped below this threshold after *BRCA*-result disclosure; no clinically relevant distress scores were shown in DNA-direct. Heredity-specific distress (IES-her) did not differ significantly between procedures, nor showed an interaction effect. BC-specific distress in DNA-direct did score lower than DNA-intake (IES-bc: *p* = 0.03) without an interaction effect. All heredity-specific and BC-specific distress scores remained below the clinical relevance threshold of IES ≤ 26.

**Table 2:** Psychological measures for all breast cancer (BC) patients choosing DNA-direct (novel format, *n* = 59) or DNA-intake (usual care, *n* = 49) responding at follow-up 1 year post *BRCA*-result disclosure (T2). Estimated means ± standard deviations are reported following correction for baseline differences (see Table 1) in repeated measurements ANOVA

Characteristic	T0		T1		T2		P*
	DNA-direct *n* = 59	DNA-intake *n* = 49	DNA-direct *n* = 59	DNA-intake *n* = 49	DNA-direct *n* = 59	DNA-intake *n* = 49	
General distress (GHQ-12: 0–12)	2.7 ± 3.0	4.2 ± 3.5	1.9 ± 3.0	3.9 ± 3.5	1.8 ± 3.0	2.1 ± 3.5	***0.01*** **
Heredity specific distress (IES-her: 0–75)	13.9 ± 14.5	15.1 ± 14.9	12.3 ± 16.0	14.9 ± 16.2	9.1 ± 12.9	13.4 ± 13.0	*0.26*
BC-specific distress (IES-bc: 0–75)	17.0 ± 15.7	23.7 ± 15.8	*not measured*	*not measured*	12.4 ± 14.2	18.1 ± 14.4	***0.03***
Quality of Life (QoL: 0–100)	73.1 ± 20.0	71.9 ± 20.1	75.0 ± 16.9	73.5 ± 17.3	77.9 ± 19.2	77.7 ± 19.4	*0.76*
BC worry (CWS: 8–32)	14.1 ± 3.8	15.2 ± 4.0	14.5 ± 3.0	15.2 ± 3.3	13.5 ± 3.8	14.8 ± 3.3	*0.10*
Risk (0–100) perception: hereditary BC	40.4 ± 24.7	41.1 ± 25.2	32.8 ± 29.9	32.4 ± 30.5	38.6 ± 29.2	38.7 ± 29.8	*0.98*
Risk (0–100) perception: 2nd BC	45.4 ± 29.2	44.1 ± 29.5	37.7 ± 24.7	41.8 ± 24.9	38.9 ± 28.4	43.2 ± 28.9	*0.62*

* Reported *P*-values are associated with the main effect for choice of procedure (DNA-direct versus DNA-intake) in repeated measurements ANOVA: bold indicates statistical significance *p* < 0.05. No main effects for time (within subjects) were found

** Trend for interaction effect between time and choice of procedure: *p* = 0.051. No other variables showed (trends for) interaction effects

Variables significantly correlating with general distress (GHQ-12), heredity-specific distress (IES-her) or BC-specific distress (IES-bc) are shown in Table 3. Choice of procedure (DNA-direct versus DNA-intake) only correlated to heredity-specific distress, but was no longer significant following multivariate regression analysis. Higher BC-specific distress was a significant contributor to both general distress (*p* = 0.01) and heredity-specific distress (*p* < 0.001). General distress was also more likely in participants with lower quality of life (*p* < 0.001), while higher heredity-specific distress was associated with more BC worry (*p* = 0.01) or having a sister with BC (*p* = 0.02). More BC-specific distress was seen in those with higher heredity-specific distress (*p* < 0.001), higher BC worry (*p* < 0.001) or younger age at inclusion (*p* = 0.02).

**Table 3:** Determinants of psychological distress measures amongst all participating breast cancer (BC) patients choosing either DNA-direct (intervention) or DNA-intake (control), following correlation testing with choice of procedure, sociodemographics, BC characteristics and other psychological variables (significant correlations with *p* < 0.05 shown)

Characteristic	Pearson’s correlation coefficient	Multivariate regression analysis		
		P	Beta	95 % confidence interval
General distress (GHQ-12)				
- Quality of Life (QoL)	−0.562	<0.001	−0.544	[−0.114 – -0.063]
- BC-specific distress (IES-bc)	0.255	0.011	0.205	[0.010–0.073]
- BC worry (CWS)	0.275	n.s.	–	–
- mother with BC	−0.363	n.s.	–	–
- age youngest relative with BC	−0.428	n.s.	–	–
- children living at home	0.196	n.s.	–	–
Heredity-specific distress (IES-her)				
- DNA-direct vs. DNA-intake	−0.192	n.s.	–	–
- BC-specific distress (IES-bc)	0.545	<0.001	0.386	[0.190–0.600]
- BC worry (CWS)	0.520	0.013	0.257	[0.239–1.983]
- sister with BC	0.206	0.022	0.183	[0.929–11.723]
BC-specific distress (IES-bc)				
- General distress (GHQ-12)	0.255	n.s.	–	–
- Heredity-specific distress (IES-her)	0.545	<0.001	0.304	[0.135–0.458]
- BC worry (CWS)	0.631	<0.001	0.452	[1.210–2.618]
- age at inclusion (yrs)	−0.233	0.024	−0.165	[−0.411 – -0.030]

### Secondary Psychological Outcomes

Quality of life, BC worry, risk perception for hereditary BC and 2nd BC (Table 2) did not differ between DNA-direct versus DNA-intake (between subjects) or over time (within subjects).

### Perceived Causes of Breast Cancer

Participants were asked what they thought may have caused their breast cancer as an open-ended question. As multiple reasons were possible per responder, percentages are not cumulative: heredity (30 %), bad luck (30 %), stress (14 %) and hormonal factors e.g., oral contraception (14 %) were reported as perceived causes of BC; 18 % did not know. Only one significant difference was found: those in the DNA-direct group were more likely to perceive heredity (e.g., “it runs in the family”) as the cause of their BC than in DNA-intake (46 vs. 10 %, *p* < 0.001).

### Important Aspects of Genetic Testing

Participants were also asked what they thought was important to know for other patients who may be eligible for BC genetic testing as an open-ended question. Responders felt (not cumulative) that these other patients should know about: certainty and/or clarity about a hereditary predisposition (22 %), consequences for family (18 %), procedural aspects (18 %), consequences of genetic testing (18 %), early prevention (12 %) and no full guarantees from test results (11 %). No differences between DNA-direct and DNA-intake were found.

### Genetic Counselors’ Experiences

From reports of the DNA-direct disclosure sessions (*n* = 88), genetic counselors were able to establish good rapport with patients despite not having spoken to them previously (94 %), (94 %), experienced few unexpected patient reactions (7 %), few patients needing follow-up consultations (9 %) or non-standard psychosocial support (2 %), believed most patients made an informed choice to start *BRCA*-testing (76 %) and in retrospect, did not prefer a pre-test intake consultation for the majority of patients (85 %). In general, counselors reported the benefit of to-the-point and personalized counseling, saving time within the 45 min of a first face-to-face consultation to discuss personal consequences of the known *BRCA*-result for the patient and her family.

## Discussion

The current evaluation continues our previous study, which had already shown that more patients with BC chose the new format of *BRCA*-mutation testing without prior face-to-face genetic counseling (DNA-direct) over the current standard of face-to-face counseling both prior to and following *BRCA*-mutation testing (DNA-intake). Follow-up of these patients showed that there was no increase in psychological distress at either short- or long-term. In fact, the DNA-direct participants scored lower on both general and BC-specific distress than DNA-intake, although distress scores remained below the level of clinical relevance in both groups. We conclude from these study results that the DNA-direct procedure i.e., face-to-face counseling after availability of *BRCA*-mutation testing results, is appropriate especially for BC patients who are similar to DNA-direct participants in our study. Patients without pre-existing psychological problems may prefer to arrange *BRCA*-mutation testing from home due to reasons associated with their BC diagnosis and treatment, information needs and preferences, as well as certain family characteristics (e.g., children living at home). In retrospect genetic counselors did not prefer DNA-intake for most patients (85 %). Counselors emphasized the value of to-the-point and personalized counseling with more time to discuss personal consequences rather than general *BRCA* information. Counselors also reported that most patients did not require additional follow-up (91 %).

One interesting difference at long-term follow-up should be noted: more DNA-direct participants reported their belief that heredity (may have) caused their BC, but this was not reflected in reports of risk perception for hereditary BC, which remained equal between groups and over time. However, one of the baseline differences found between groups was that amongst DNA-direct participants, BC was diagnosed in their family at a younger age. Although no other clinical variables were previously found to predict for the choice of DNA-direct (Sie et al. 2014a) this may suggest that these participants are more aware that their BC risk may still be moderately increased by familial factors, beyond *BRCA*-mutations. Risk perceptions for hereditary BC not changing over time for either group is also notable and in concordance with earlier literature showing that traditional genetic counseling and testing has no lasting effects on risk perception (Braithwaite et al. 2006; Hilgart et al. 2012). Improving patient risk perceptions remains a challenge for genetic counseling as a whole, but is not enhanced nor deteriorated due to the DNA-direct procedure.

However higher uptake of *BRCA*-testing (100 % in DNA-direct versus 76 % in DNA-intake) might suggest more patients were *BRCA*-tested unnecessarily in DNA-direct: *BRCA*-testing was only indicated if one or more of familial risk scores (e.g., FHAT (Gilpin et al. 2000), Myriad (Frank et al. 2002), Claus/van Asperen (van Asperen et al. 2004)) exceeded certain thresholds. But familial risk selection criteria for *BRCA*-testing were not fulfilled by some patients *BRCA*-tested in both groups: 35 % in DNA-direct versus 26 % in DNA-intake. Mutation detection rate remained equal in both groups. This reflects that the choice of procedure did not result in different numbers of patients *BRCA-*tested, whereas offering DNA-direct alongside DNA-intake increased patient participation and reduced processing time. We consider this to be the greatest benefit of the DNA-direct procedure.

### Study Limitations

As described previously (Sie et al. 2014a), non-randomization of our study participants limits the ability to argue cause/effect while non-random sampling limits the generalizability of our study results. DNA-direct may therefore be most appropriate for those BC patients matching the overall profile of DNA-direct participants in our study: those who are higher educated and better informed, as well as comfortable with or even preferring different information formats beyond face-to-face contact. We now think that randomization for our study would be unethical, as our study results suggest a link between distress and self-selection; although the study limitations remain. Another study limitation is the low number of *BRCA*-positive results which may have influenced our study results, as these patients are the most likely to experience distress after disclosure (Nelson et al. 2014). However, this low number is reflective of standard clinical genetic practice therefore does not affect generalizability of our study results. Nearly a third of our original study cohort did not complete currently reported long-term follow-up measures, which may also have influenced results.

No formal cost-effectiveness analyses have been performed, but DNA-direct reduced face-to-face consultation time for both genetic counselors and the surrounding resources at the outpatient clinic. The DNA-intake procedure included a pre-test session of 45 min and a post-test session of 15 min (total 60 min). The DNA-direct procedure only included a post-test session of 45 min. We consider such reduced consultation and processing times, as well as the increased patient participation of the DNA-direct procedure, to outweigh cost-effectiveness not formally being proven, dependent on additional *BRCA*-test costs.

Finally, other events could cause distress in our BC patient population: time is the only trigger assessed in this study. However our main interest was the general trend of distress between the two procedures: other triggers for distress could be equally present in either group and were not expected to influence study results.

### Practice Implications

Participants who chose the traditional DNA-intake procedure reported higher general and BC-specific distress, even 1 year after *BRCA*-result disclosure. This supports our earlier notion (Sie et al. 2014a) that distressed patients were more likely to choose face-to-face counseling prior to genetic testing. However, choice of procedure did not appear to be a significant contributor to general and heredity-specific distress, instead both were associated with BC-specific distress. This further suggests that higher distress scores were based on the experience of BC, not the (chosen) genetic diagnostic procedure; and that those who feel more distressed and may be in need of prior psychosocial support, self-selected to the DNA-intake procedure where such support was immediately available. Offering DNA-direct as an alternative to the standard DNA-intake, to match individual preferences for information formats prior to *BRCA*-mutation testing, therefore is considered acceptable in the light of our follow-up results. This adds to an ever-growing body of literature (Albada et al. 2011; Butrick et al. 2014; Metcalfe et al. 2010; Schwartz et al. 2014; Voorwinden et al. 2012) showing that these new models of cancer genetic services varying in combinations of face-to-face, telephone and/or digital communication, pre- and/or post *BRCA*-testing, are acceptable (Trepanier and Allain 2014). Positive patient experiences with newer multi-gene panels (Sie et al. 2014b) have currently only been proven after pre-test counseling regarding possible unsolicited or unclear findings (Rigter et al. 2014). Therefore we do not currently recommend DNA-direct for multi-gene panels.

### Research Recommendations

Other target groups for DNA-direct may be evaluated. For example, *BRCA*-mutations account for 5–16 % of all ovarian cancer cases (Ramus and Gayther 2009) and guidelines now recommend referral of all patients with ovarian cancer regardless of age or family history (Netherlands 2012). Patients with ovarian cancer strongly supported genetic testing around the time of diagnosis (Meiser et al. 2012) and may be excellent candidates for DNA-direct in the future. Further research may also focus on alternative service models for the multi-gene panel setting, starting with those now used for conventional single-gene testing (Trepanier and Allain 2014).

## Conclusions

BC patients who had chosen to forego personal genetic counseling prior to *BRCA*-mutation testing, and instead receive a combination of telephone, written and digital information reported high satisfaction and low distress both several weeks and 1 year after *BRCA*-result disclosure. Distress in this population appears to be triggered by the BC diagnosis, not genetic testing. The novel DNA-direct procedure appears acceptable for BC patients alongside the traditional face-to-face intake procedure.
